# Dermatology and Syphilis

**Published:** 1876-10

**Authors:** 


### IV. Dermatology and Syphilis.

1. *Tracheotomy in Extremis for Asphyxia from Syphilitic Laryngitis.* Quise. (*Lyon Médic.*, No. 26.)

A patient with syphilitic antecedents had secondary laryngeal disorder, with subsequent complete aphonia and dysphagia. Suddenly the hoarseness, which remained after the aphonia was relieved, became aggravated, and asphyxia rapidly supervened. After two of these attacks he resorted to the hospital, and, on the night following his entry, a third attack produced insensibility. At 10 A. M. he was apparently dead—respiration was suspended, the face was turgescent and cyanosed, pulse imperceptible, insensibility absolute. Tracheotomy was performed with a single stroke, a double cannula inserted, and respiration gradually established, the patient during his restoration exhibiting convulsive phenomena of the face, limbs and trunk. One hour elapsed before he was restored to consciousness. An abundant purulent discharge escaped from the cannula for about two weeks, producing much disturbance of respiration, sleep, deglutition, etc.; but under the influence of the iodide of potassium this gradually diminished, and finally ceased, when the cannula was removed.

The laryngoscopic examination disclosed several white spots upon the vestibule of the glottis, a normal epiglottis, and thickened vocal cords, the right showing several small whitish points, with three small indentations of a light rosy hue, upon the border.

A second examination, twelve days later, disclosed a marked improvement in all these particulars. The patient was entirely relieved, except for the moderate vocal huskiness.

2. *Diagnosis of Pustulo-Crustaceous Syphilide.* Hardy. (*Le Progrès Méd.*, No. 33.)

The distinguished professor, in his clinical remarks upon the case of a woman affected with this disease, remarked that in scrofula the ulcerations are covered with a more voluminous crust, and have a special color. They are white or gray, or even black if a small amount of blood has been effused—never yellowish-green, as in this instance. In scrofula, also, there is œdematous swelling of the tissues in the neighborhood of the ulceration. When upon the face, the nose is increased in size, the upper lip is thick and projecting. In this instance there is, it is true, a slight degree of swelling of the skin, but it is dense and firm. Here also the tint is brownish or dark brown—it is not of a vinous color, as in scrofula. Besides, the lesions of scrofula are slow of evolution, requiring months and even years. It is rare that pustules are produced and cured as rapidly as in this case. Here the patient has almost had no treatment, and yet the disease has disappeared from the nose. If we had to deal with a scrofulide, we should find at this point ulceration and crusting in full activity.

Here also there is no history of scrofula. It is exceedingly rare that crofulous manifestations appear upon the skin for the first time after the thirtieth or fortieth year, and even in that case there has generally been some predisposing accident to the tissues. The cicatrices seen upon the limbs of the patient are not scrofulous; they are characteristic of syphilis —small, shallow and smooth, very slightly depressed. The cicatrices of scrofulides are either projecting or deeply depressed, reticulated, and often present the appearance of keloid.

3. *Biniodide of Mercury in Superficial Lupus.* GUIBOUT. (*Gaz. des Hôpit.*, Aug. 5, 1876.)

A female confectioner, thirty-seven years old, had upon the chin and the larger part of the left cheek a superficial lupus, or tuberculo-ulcerative scrofulide, which had lasted for fifteen years. No good results had been obtained from various local applications, including the actual cautery. The affection almost completely disappeared after five applications of an ointment consisting of equal parts of lard and the biniodide of mercury. The cicatrices are still somewhat red, but are gradually losing this color. She stated that each application produced, about one hour afterward, an almost intolerable pain, which lasted for five or six hours. At the same time a great quantity of "reddish water" escaped from the surfaces attacked. The author states that the ointment produces a crop of ecthymatous pustules, which burst and form crusts. When these latter separate the skin beneath is found less swollen and more supple, while the ulcerated patches have begun to cicatrize.

The applications should not be too frequently repeated. Eight days after the first the patient referred to above was in an improved condition. The lupus itself had ceased to be painful. The cheek grew smaller, its normal volume was restored, cicatrization rapidly ensued, and is now perfect. Upon the skin nothing remains but a few rows of minute tubercles, upon which a layer of ointment has been spread.

4. *Syphiloma of the Tubercula Quadrigemina.* FIORI. (*Gaz. di R. Accad. di Med. di Torino*, Aug., 1876.)

The author reports the case of a young man, with probable syphilitic antecedents, who had a saber wound of the left parietal region, from which he recovered. Soon after he fell to the ground insensible, but was relieved after suffering from transient hemiplegia. Then followed peculiar spasmodic accesses, with decreasing intervals, in which the head was twisted to the left, and the entire body performed partial rotations to the left upon its vertical axis. In the later attacks there was no loss of sensibility, but the eyes were turned to the left, and the tongue, left labial commissure, uvula, pupils and sterno-cleido-mastoid muscles were manifestly affected.

The author analyzes the meaning of the various symptoms in the case, and compares them with the results obtained by the experiments and researches of Adamuk, Schiff, Ferrier, Hitzig, Fritsch, Brown-Sequard and others. He concludes that there was lesion of the tubercula quadrigemina, with possible arterial complication (especially at the outset), and localized hæmorrhage. The treatment justified the diagnosis—at least in part. No improvement was had by the administration of bromide of potassium and chloral, or by hypodermic injections of sulphate of atropia. Complete cure was obtained after employment of mercury and the iodide of potassium, the patient leaving the clinic of the Royal University of Turin in a condition of perfect health.

